# Fluorescent nuclear track detectors for out‐of‐field neutron dosimetry in proton therapy

**DOI:** 10.1002/mp.70303

**Published:** 2026-02-10

**Authors:** Stefan Schmidt, Iván D. Muñoz, Eduardo G. Yukihara, José Vedelago

**Affiliations:** ^1^ Department of Radiation Oncology Heidelberg University Hospital (UKHD) Heidelberg Germany; ^2^ Division of Medical Physics in Radiation Oncology German Cancer Research Center (DKFZ) Heidelberg Germany; ^3^ Heidelberg Institute for Radiation Oncology (HIRO) and National Center for Radiation Research in Oncology (NCRO) Heidelberg Germany; ^4^ Medical Faculty Heidelberg Heidelberg University Heidelberg Germany; ^5^ Department of Radiation Safety and Security Paul Scherrer Institute (PSI) Villigen Switzerland

**Keywords:** fluorescent nuclear track detectors, neutron dosimetry, proton out‐of‐field measurements

## Abstract

**Background:**

Secondary neutrons are a major concern regarding side effects in ion beam therapy because they contribute to the out‐of‐field dose, particularly important for sensitive patient groups such as pregnant and pediatric patients. Measuring these neutrons is challenging because of their high kinetic energy, which is imparted to charged particles like fragments and recoil protons. In addition, accurate measurements require small detectors that ideally do not disturb the radiation field when measuring inside a phantom. Fluorescent Nuclear Track Detectors (FNTDs) have already shown promising results in ion beam dosimetry and the measurement in fast neutron fields. Given their high spatial resolution and sensitivity, FNTDs offer a promising approach for characterizing secondary neutron doses in complex radiation environments, such as those encountered in proton therapy.

**Purpose:**

Establish a methodology for estimating neutron‐induced out‐of‐field dose inside a phantom. The focus is to discuss the technical requirements and present initial experimental results from a proton treatment plan.

**Methods:**

The analysis workflow for determining dose equivalent with FNTDs is introduced, including intensity‐to‐linear energy transfer (LET) in water conversion and track polar angle corrections. FNTDs were placed inside RW3 and polymethyl methacrylate phantoms and irradiated with a proton spread‐out Bragg peak (SOBP) plan. Experimental results from two downstream positions in each phantom were used to benchmark Monte Carlo simulations.

**Results:**

A polar angle correction function was established, indicating intensity corrections of approximately a factor of 2 at 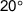 and up to a factor of 3.5 beyond 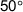. Furthermore, a few short‐range high‐LET tracks with a low probability of occurrence have been found. Despite accounting for only about 1% of the total fluence, high‐LET tracks can contribute more than 50% of the total dose equivalent. When not considering these short‐range tracks, the relative agreement in dose equivalent between simulations and experiments was within (1.10±0.10) to (1.49±0.13).

**Conclusions:**

This work presents the first LET‐based method using FNTDs to estimate out‐of‐field neutron dose for a proton SOBP plan, measured inside a phantom. Integrating this method into clinical workflows may improve out‐of‐field dose estimation for sensitive patient groups, such as pregnant or pediatric patients, by enabling prior dose assessments using anthropomorphic phantoms.

## 1 INTRODUCTION

In ion beam therapy, one of the main concerns regarding side effects is the risk of secondary tumors induced by out‐of‐field doses, which is of special importance to pediatric and pregnant patients.^1^ The out‐of‐field dose in proton beam therapy can be divided into photon and neutron contributions.^2^, ^3^ Whereas the photon contribution, mainly due to prompt gammas from excited nuclei, can be measured quite accurately with widely established detectors, such as radiophotoluminescent and thermoluminescent detectors, the neutron contribution is more difficult to measure.^4^, ^5^, ^6^ This becomes even more challenging when measurements inside a phantom are needed to determine the dose at a certain position. For example, in the case of a fetus, such measurements are required to estimate the additional risk if a pregnant patient needs radiotherapy. This imposes requirements on the detectors, including a small size to fit into the desired position inside the phantom without disturbing the radiation field.

In previous studies, the main candidates for neutron dose estimation inside a phantom were Plastic Nuclear Track Detectors (PNTDs) or bubble detectors.^4^, ^5^, ^6^, ^7^ While PNTDs are small and insensitive to photon dose, they require dedicated post‐processing steps, such as chemical etching.^8^ Moreover, PNTDs have a relatively low detection efficiency for particles with a linear energy transfer (LET) in water below $10\;\mathrm{keV}\mu m^{-1}$, limiting the detection of secondary particles such as recoil protons to energies of a few MeV.^9^ Bubble detectors introduce a rather large volume to the phantom, thus potentially disturbing the field to be measured and not allowing for measuring with a high spatial resolution.^10^, ^11^

It has been shown that Fluorescent Nuclear Track Detectors (FNTDs) based on alumina doped with carbon and magnesium (${\mathrm{Al}}_{2}O_{3}$:C,Mg) can measure charged particle tracks down to LET values of $0.4\;\mathrm{keV}\mu m^{-1}$, allowing them to detect protons up to 220MeV.^12^, ^13^, ^14^ Moreover, with a typical dimension of 8.0mm × 4.0mm × 0.5mm, they can be easily placed inside a phantom. Additionally, FNTDs do not require any special processing after readout and can be read out directly with a confocal laser scanning microscope. However, FNTDs have been mainly utilized for neutron dosimetry of ambient or personal dose equivalent, which are not suitable quantities to report dose at the position of measurement inside a phantom.^15^, ^16^, ^17^

The goal of this study was to develop a methodology for dose equivalent estimation based on particle LET. This involved implementing 3D track analysis, correction methods for measuring track angle and intensity, and a conversion function to correlate track spot intensity with particle track LET. The accuracy of the developed method for out‐of‐field dose estimation was benchmarked using a spread‐out Bragg peak (SOBP) proton treatment plan. Measurements were performed at two positions behind the SOBP in two different phantoms. The results were compared with Monte Carlo (MC) simulations.

## 2 MATERIALS AND METHODS

### 2.1 Experimental setup and irradiations

**FIGURE 1:**
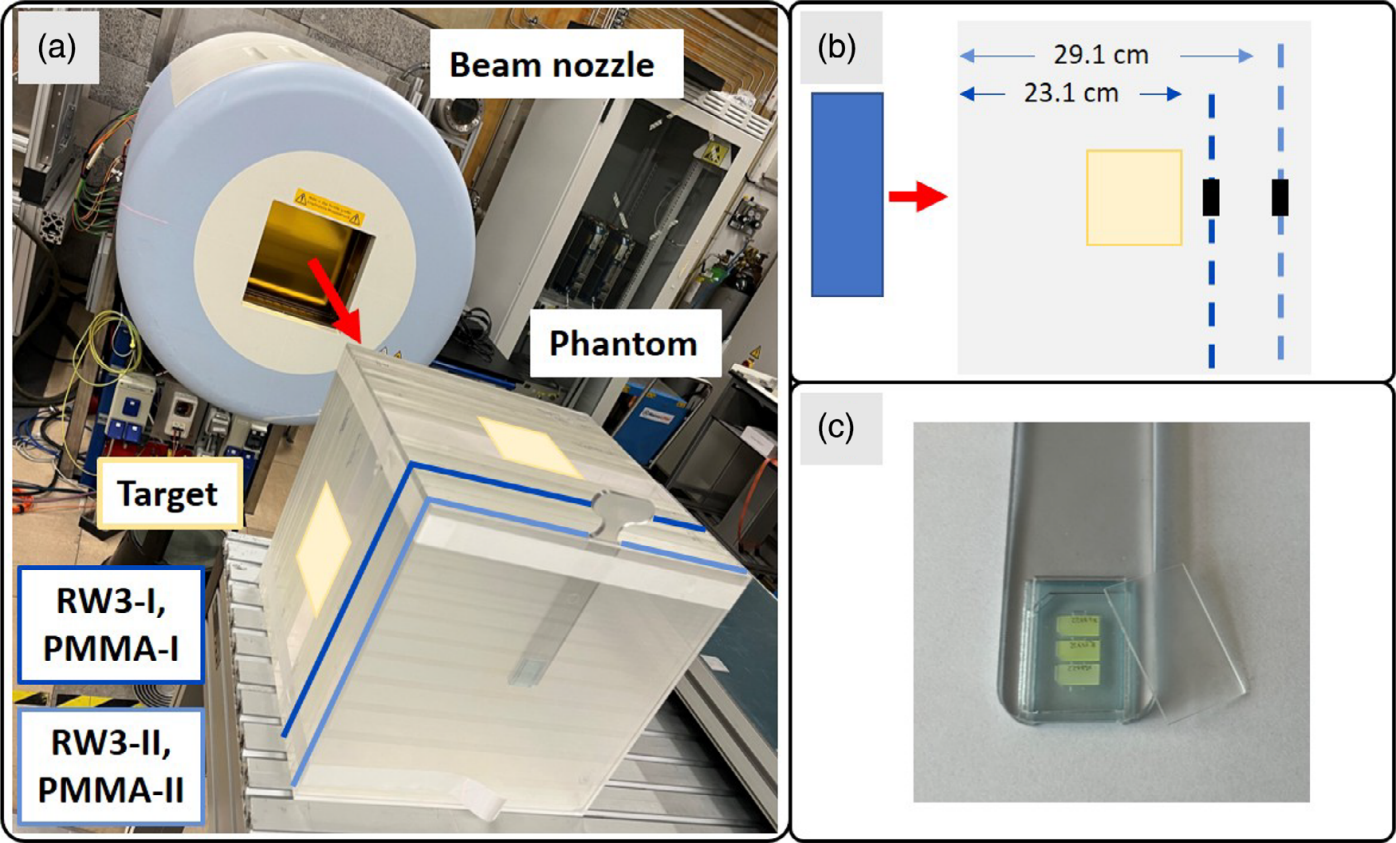
Visualization of the experimental setup for in‐phantom measurements. (a) Beam nozzle and the polymethyl methacrylate (PMMA) phantom with the two different measurement positions at 23.1cm in dark blue for RW3‐I and PMMA‐I and at 29.1cm in light blue for RW3‐II and PMMA‐II. The yellow areas indicate the orthogonal projections of the target volume on the phantom surface. (b) Schematic top view of the detector positions inside the phantom behind the target. (c) Close‐up view of the 3D printed holder with three Fluorescent Nuclear Track Detectors (FNTDs) positioned next to each other. The material of the 1mm thick cover matched the phantom material.

The experiments were conducted at the experimental room of the Heidelberg Ion Beam Therapy Center (HIT; Heidelberg, Germany), where a horizontal beamline is available.^18^ For the experiments, a simplified 30cm × 30cm × 30cm slab phantom was used, as depicted in Figure 1a. Two different materials for the phantom were employed: polymethyl methacrylate (PMMA) and RW3. The detectors were positioned by utilizing a 3D‐printed holder downstream of the isocenter and outside of the target volume, with the normal of the detector parallel to the beam (the material specifications are listed in Section S1).

For the irradiation, a squared proton SOBP field was applied, containing primary protons with energies ranging from $118.78\;\mathrm{MeV}\;u^{-1}$ to $171.60\;\mathrm{MeV}\;u^{-1}$, as listed in Section S2. The irradiation plan was designed for a same‐sized phantom but with water as material, yielding a target volume of 10cm × 10cm × 10cm, centered at the isocenter, as used in previous out‐of‐field studies.^5^, ^19^ Dose measurements were performed distal to the target volume at two depths inside the phantom, as depicted in Figure 1b. The measured setups and the delivered target dose values are reported in Table 1. The proximal position is indicated in dark blue with the label “I”, and the distal one is highlighted in light blue with the label “II”. The prefixes “RW3 ”and “PMMA” indicate the phantom material used. For each measurement point, three FNTDs were irradiated simultaneously, as visualized in Figure 1c.

**TABLE 1:** Overview of the measurement conditions for each setup and corresponding phantom material, including the depth of the measurement position (measured from the proximal surface of the phantom) and the target dose.

Setup	Phantom material	Position in phantom / cm	Target dose / Gy
RW3‐I	RW3	23.1	3
RW3‐II	RW3	29.1	3
PMMA‐I	PMMA	23.1	2
PMMA‐II	PMMA	29.1	3

### 2.2 FNTDs

FNTDs made of alumina are manufactured by Landauer Inc. Crystal Growth Division (Stillwater, OK, USA) and were employed for the measurements.^12^ They are polished to optical quality on one side, which represents the front of the detector.

To address inter‐detector sensitivity variations, irradiations with a 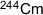 alpha source were conducted, following a methodology previously reported and validated for in‐field LET measurements in clinical proton beams.^14^, ^20^ By analyzing the track intensities from a source with a constant radiation quality, variations attributed to the detector sensitivity can be detected. The 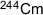 irradiations were restricted to the central region of the detectors (between 3mm and 5mm), with the remaining area shielded by the holder. With a 75s exposure and a readout area of $0.0049\;\;{\mathrm{cm}}^{2}$, approximately 1000 tracks were analyzed per detector.

The detector readout was conducted using a confocal laser scanning microscope dedicated to FNTD readout (FXR700RG, Landauer Inc.).^21^ Example microscope images are shown in Section S3. Each detector was scanned in 3D with 11 slices in depth, starting at a depth of 2μm and an interval of 2μm between each slice. For each image, a readout time of 10s (equivalent to 38μs dwell time) and a size of 100μm × 100μm with 512pixels×512pixels was chosen. For each detector, 125 images were taken to analyze the out‐of‐field signal induced by neutrons, corresponding to a total scanned area of approximately $0.0122\;\;{\mathrm{cm}}^{2}$. Additionally, 50 images were acquired to determine and correct for detector sensitivity variation. These images contained both neutron‐induced tracks and tracks from the 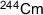 alpha source. The latter ones were not only used to address inter‐detector sensitivity variation, but also to address changes in the performance of the confocal microscope. In these images, the alpha tracks resulted in a dominant signal, and the overlapping out‐of‐field tracks were filtered, differentiating by the particle range, which is not greater than 16μm for the alpha particles in alumina.^14^

For the detector evaluation, an in‐house MATLAB post‐processing methodology was used, based on previous work.^17^ The previous 2D track identification was now extended to 3D by implementing a track spot linking procedure. The 3D methodology simultaneously reveals information about the track polar angle (which represents the angle between the impinging ion track and the normal of the detector surface) and the track intensity, which can be related to the LET of the corresponding ions (the LET calibration curve is reported in Section S4). In addition, the 3D analysis enables better discrimination between ion and delta electron tracks, which is necessary because high‐energy proton and delta electron tracks can produce similar intensities, thereby complicating track spot discrimination in 2D. However, in this work, delta electrons were typically only present in three to four consecutive microscope images (about 4μmto6μm).

### 2.3 MC simulations

MC simulations were performed using the latest FLUktuierende KAskade (FLUKA) version, as reported in Section S5. The simulation input files were linked to event generators to perform interactions above $125\;\mathrm{MeV}\;u^{-1}$. The Beam Accelerator Monitoring System (BAMS) was implemented according to a previous study.^22^ The phantoms were modeled by a 30cm × 30cm × 30cm cube made either of PMMA or RW3. The materials used in the simulations are reported in Section S2. The whole setup was surrounded by a concrete box of 30cm thickness, resulting in inner room dimensions of 6.5m × 8.0m × 3.5m, similar to the experimental room at HIT, without considering any other body of the room, as done previously.^19^ For the FNTDs, the phantom material used was assigned to record only the signal coming from the phantom and not from the alumina. The scoring of charged particles was performed using the mgdraw user routine, which scored each charged particle crossing the scoring plane, together with other parameters such as track angle and LET. For the scoring, a planar area of $1\;\;{\mathrm{cm}}^{2}$ was used. With this information, the fluence was determined by counting the number of crossing particles per area, not taking into account the angle of the particles. For the MC simulations, the uncertainty was estimated based on batch statistics, as implemented in the FLUKA scorers.

### 2.4 Track angle intensity correction

In previous work, a decrease in track spot intensity with increasing track polar angle θ has been reported for FNTDs.^23^ However, this relationship was modeled analytically and has not yet been validated with experimental data to the best of the authors' knowledge. Since accurate estimation of intensity and thus LET is required to calculate dosimetric quantities, this relationship was further investigated in this work for the FXR700RG reader. In particular, for out‐of‐field measurements, greater track angles are expected, which further motivates the need for this analysis.

A total number of three FNTDs were exposed to mono‐energetic carbon ions at HIT with an energy of $430.10\;\mathrm{MeV}\;u^{-1}$. The carbon ions were chosen to ensure that even tracks with polar angles up to 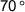 can be properly identified. Each FNTD was exposed to four nominal angles with respect to the normal of the FNTD surface, either {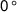; 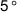; 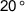; 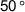}, {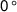; 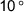; 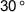; 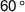}, or {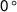; 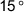; 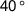; 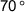}. Inter‐ and intra‐detector sensitivity variation was corrected by normalizing the track intensities with the intensities of tracks at an angle of 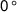. To realize these polar angles, the FNTDs were mounted on a 15cm × 15cm PMMA block with their front side facing the beam, and the PMMA block was rotated to the desired angle. For this analysis, 36 images with a total area of $0.0035\;\;{\mathrm{cm}}^{2}$ were read out per detector, with 10 slices in depth.

In the post‐processing, overlapping tracks were rejected to avoid false intensity assignments. The track intensity was calculated as the average of spot intensities from the second to the penultimate slice, where the maximum intensity within the spot represented each slice's value. In the first step, a fourth‐order Gaussian function was fitted to the distribution of track angles, binned in $1^{\circ }$ intervals, to determine the average track angle for each of the four irradiations in every FNTD. In the second step, the intensity values of tracks with a polar angle within the range of the mean ±1σ were compiled into a histogram using a bin size of 100 a.u. A first‐order Gaussian function was then fitted to these intensities to determine the average intensity value for each measured angle. Finally, these intensities were normalized to the mean intensity of tracks with a polar angle of $0^{\circ }$ for each detector. The relationship between track polar angle and the reduction of track spot intensity y(θ) was represented by a logistic function, with an intensity value of 1 for $0^{\circ }$, and a converging intensity value for large angles, being close to 0.3, as suggested in literature.^23^ To describe the experimentally obtained data with a semi‐empirical model, the logistic function according to Equation 1 was fitted by utilizing the nonlinear least squares method:

(1) $$y\left(\theta \right)=\frac{a}{1+e^{b\cdot (\theta -c)}}+\left(1-\frac{a}{1+e^{-b\cdot c}}\right)$$

where *a*, *b*, and *c* are fitting parameters. The second term in Equation 1 is a constant offset that ensures y(θ=0)=1. This polar‐angle–dependent correction factor was applied to each track to determine the intensity it would have at $0^{\circ }$, so that the LET calibration curve obtained at $0^{\circ }$ can be applied.

### 2.5 Dose equivalent estimation

The absorbed dose D was calculated using the unrestricted LET and the particle fluence, considering θ. For the range‐restricted comparison with the simulations, as used in this study, only charged particles were considered whose energy is sufficient to reach 10μm depth, taking into account the tracks' polar angle, as elaborated in Section S6. This threshold was set to allow proper identification of the track over five slices in the experimental data and to account for the energy loss straggling of the particle. D was calculated as:

(2) $$D=\frac{1}{\rho \cdot A}\sum_{i=1}^{N}\frac{{\bar{\text{LET}}}_{i}}{\cos\theta_{i}},$$

where ρ is the density in units of $g\;{\mathrm{cm}}^{-3}$, and A is the surface area of the detector being analyzed in $\;{\mathrm{cm}}^{2}$, analogous to previous definitions for other track detectors.^8^, ^24^ Average LET values along the *i*‐th track (${\bar{\text{LET}}}_{i}$) are given in units of $\mathrm{keV}\;\mu m^{-1}$. D in units of gray (Gy) was calculated by multiplying the results by a factor of $1.602\;\cdot \;10^{-9}$. The dose equivalent H in units of sievert (Sv), as defined by the International Commission on Radiological Protection (ICRP) Publication 60, was calculated as follows:^25^

(3) $$H=\sum_{i=1}^{N}D_{i}\cdot Q\left({\bar{\text{LET}}}_{i}\right),$$

where Q is a LET‐dependent quality factor, which is determined for N scored tracks. Here, the values of Q are for water, thus ρ in Equation 2 is for water. To determine the LET value for each track, the measured track intensity was corrected for the intensity decrease with track polar angle and plugged into the LET calibration curve (see Section S4).

In the comparison of simulations and experiments, a LET threshold of $25\;\mathrm{keV}\;\mu m^{-1}$ was applied. This value corresponds to the minimum kinetic energy of a proton in order to reach approximately 10μm depth in alumina, which allowed for proper discrimination of real track spots in the microscope images during post‐processing. The uncertainties were presented according to the Joint Committee for Guides in Metrology^26^ by reporting one significant digit in most cases, and two significant digits for the cases when the first digit is one.

## 3 RESULTS

### 3.1 Measured track polar angles and particle fluences agreed with simulations

**FIGURE 2:**
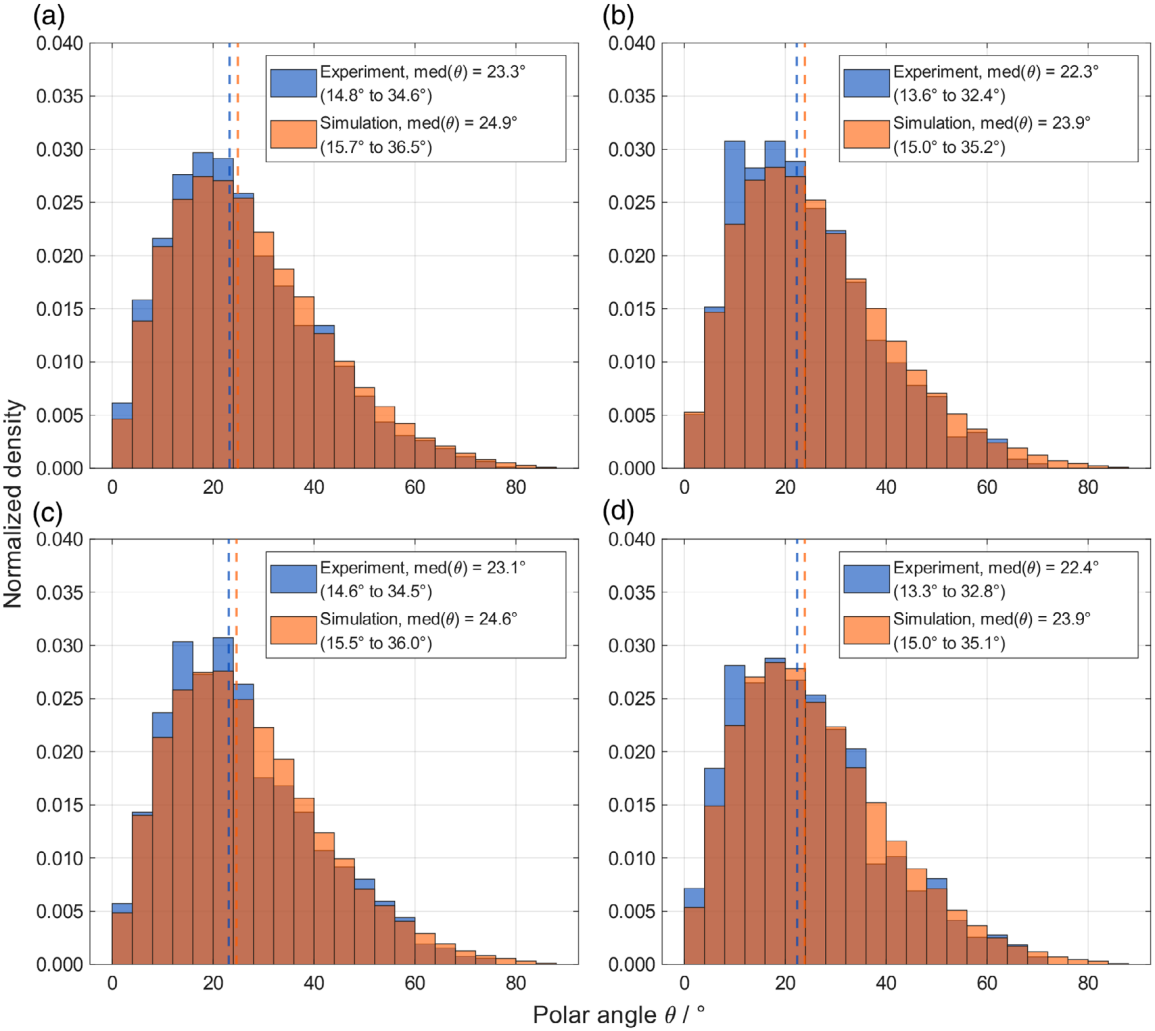
Normalized histograms of the track polar angle θ distribution for experiments (blue) and simulations (orange). Each sub‐figure corresponds to one setup, namely (a) RW3‐I, (b) RW3‐II, (c) PMMA‐I, and (d) PMMA‐II. The vertical dashed lines represent the corresponding median value of the distributions. The values in parentheses in the legend represent the interquartile range.

To evaluate the agreement between measured and simulated data, basic physical quantities were compared. Figure 2 presents a comparison between the normalized polar angle θ histograms for all four different setups. Overall, between 10%to15% of the tracks exhibited a polar angle below $10^{\circ }$. Both simulated and experimental distributions demonstrated a maximum likelihood within the range of $15^{\circ }\;\mathrm{to}\;\;25^{\circ }$. The overall angular distribution was right‐skewed. The probability of tracks with polar angles larger than the mode decreased monotonically, with fewer than 10% of the tracks exceeding $50^{\circ }$.

A slight discrepancy was apparent between the experimental and simulated distributions, as evidenced by a difference of approximately $2^{\circ }$ in the mode of the polar angle, indicated by the vertical dashed lines in Figure 2. In the legend, the median values together with the interquartile ranges of 25% and 75% are displayed, indicating agreement between simulations and experiments. Additionally, simulations revealed a larger population of tracks with polar angles exceeding $70^{\circ }$ compared to the experimental results, even though the total population of such tracks remained in the order of 1%. This discrepancy highlights the increased difficulty in detecting tracks at steep polar angles in the experiments. Between the different setups, no clear difference was observed.

Table 2 compares the experimentally obtained fluence with the one obtained with MC simulations for the four setups. For all cases, the ratios of simulated over experimental values were between 1.2to1.3. Additionally, for both materials as well as for simulations and experiments, the fluence decreased from position I to position II by about 40%to50%. The findings indicated a general consistency between experiments and simulations, as evidenced by the agreement in polar angle distribution and fluence of the particles.

**TABLE 2:** Comparison of experimental ($\phi_{\text{exp}}$) and simulated ($\phi_{\text{sim}}$) charged particle fluence, normalized by the applied target dose, for two phantom materials at two measurement positions.

Setup	$\phi_{\text{exp}}\cdot 10^{4}$ / ${\mathrm{Gy}}^{-1}$ ${\mathrm{cm}}^{-2}$	$\phi_{\text{sim}}\cdot 10^{4}$ / ${\mathrm{Gy}}^{-1}$ ${\mathrm{cm}}^{-2}$	Ratio $\phi_{\text{sim}}/\phi_{\text{exp}}$
RW3‐I	5.9±0.3	7.34±0.02	1.25±0.06
RW3‐II	3.31±0.10	4.071±0.016	1.23±0.04
PMMA‐I	5.4±0.3	6.51±0.02	1.20±0.06
PMMA‐II	2.80±0.14	3.653±0.018	1.31±0.07

### 3.2 Relevance of track intensity correction with polar angle

**FIGURE 3:**
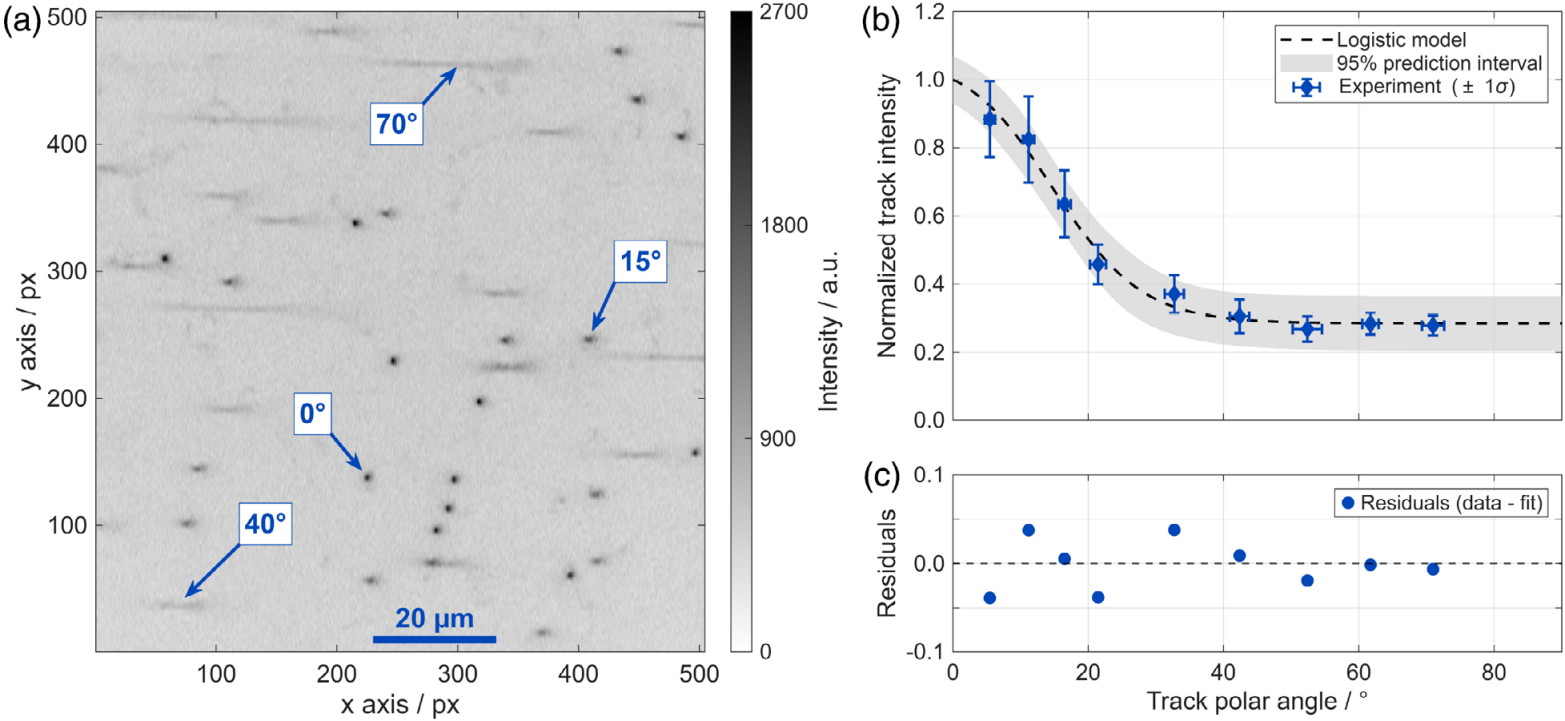
Track spot intensity analysis as a function of track polar angle. (a) Example of a fluorescence microscope image for tracks of different polar angles. The colorbar shows the gray‐scale of the intensity values. (b) Track spot intensity correction curve as a function of the track polar angle. The 95% prediction interval is highlighted in gray. The horizontal error bars display 1σ for the angle determination, and the vertical error bars represent 1σ for the intensity determination. (c) Residuals, calculated as the difference between the experimental value and the fitted value.

To quantify the decrease in track intensity with increasing track polar angle, FNTDs were exposed at nine different angles. In Figure 3a, an example image of track spots of different polar angles is shown. Tracks with a polar angle of $0^{\circ }$ have a stronger intensity and higher circularity, whereas tracks with larger angles initially become elliptical and, for polar angles exceeding $40^{\circ }$, appear as elongated lines. Here, the intensity is lower than for $0^{\circ }$ tracks. Figure 3b contains the mean track intensity values measured for the different track polar angles, normalized to the intensity of $0^{\circ }$ tracks. The error bars correspond to the 1σ standard deviation obtained from the Gaussian fits.

The quantitative results confirmed the qualitative observation in the microscope images, revealing an intensity reduction of approximately 50% for polar angles of about $20^{\circ }$. With a further increase in polar angle, the decrease in track spot intensity approached saturation, converging to a value of approximately 0.28. Since the convergence was already evident from $50^{\circ }$ onward, no relevant change was expected for track polar angles between $70^{\circ }\;\mathrm{to}\;90^{\circ }$.

The dashed line in Figure 3b represents the logistic model, introduced in Equation 1, fitted to the measured data points. The fitting parameters together with the 95% confidence interval were a=(0.79±0.11), $b={\left(0.15\pm 0.06\right)}^{\circ }$, and $c={\left(15\pm 3\right)}^{\circ }$, with a coefficient of determination of $R^{2}=0.991$. Figure 3c depicts the residuals from the fit.

Using this model, the intensity at different polar angles can be corrected, enabling a more accurate intensity‐to‐LET conversion by accounting for the strong decrease in intensity for tracks with greater polar angles. Additionally, the performance in estimating LET values was tested by analyzing FNTDs with converters that have been irradiated with mono‐energetic neutrons, as reported in Section S7.

### 3.3 Dose equivalent estimation with FNTDs

**FIGURE 4:**
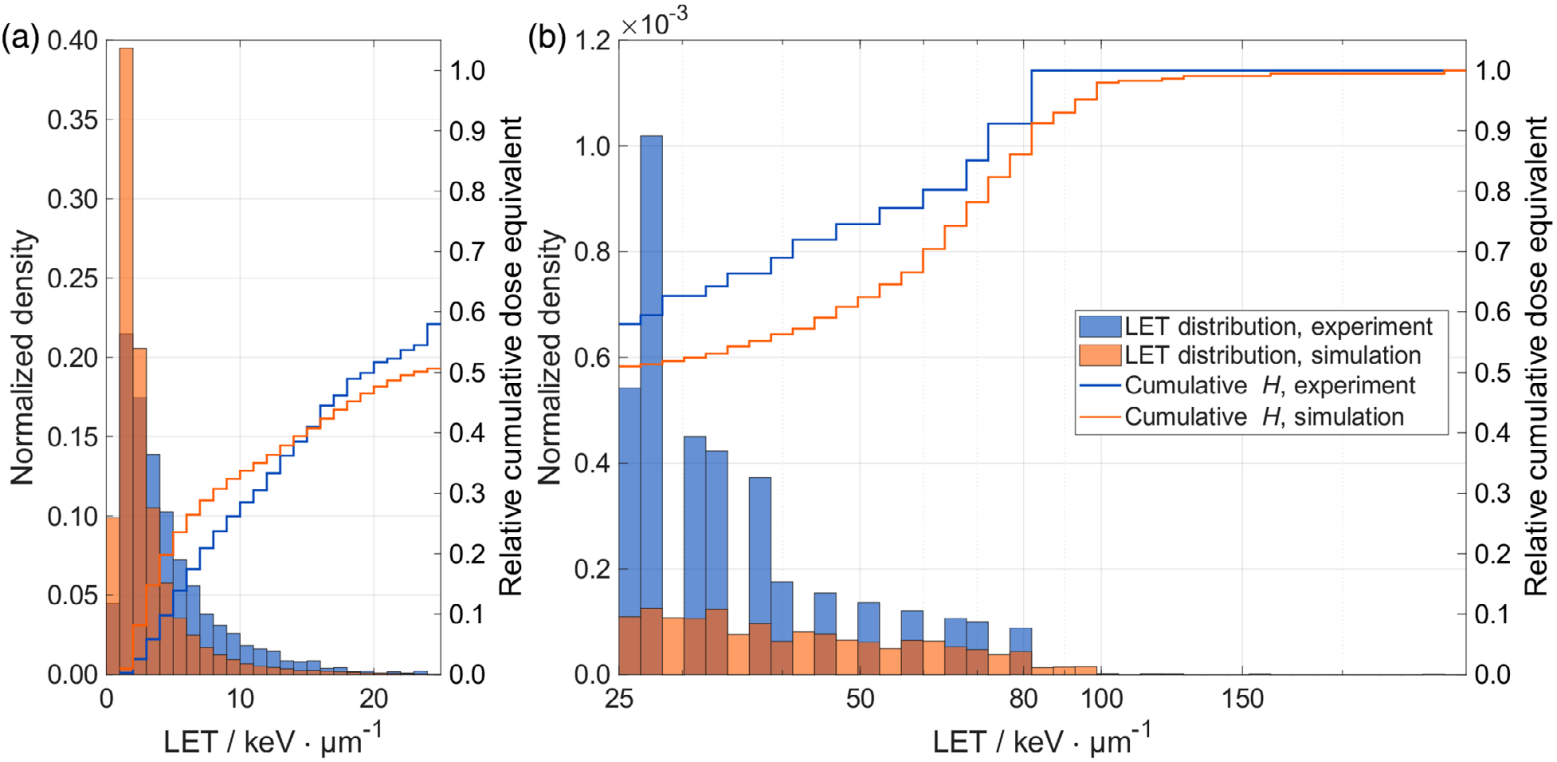
Histogram of measured linear energy transfer (LET) in water values for the RW3‐I setup. The experimental data are in blue and the simulated data are in orange. The density of each bin is indicated on the left y‐axis. The solid line shows the relative cumulative dose equivalent (H) value as a function of LET, with the corresponding values on the right y‐axis. (a) Linear binning of the data up to $25\;\mathrm{keV}\;\mu m^{-1}$. (b) Logarithmic binning of the data above $25\;\mathrm{keV}\;\mu m^{-1}$.

To evaluate the whole pipeline for the out‐of‐field dose measurement, experimental results for a proton SOBP plan were compared to MC data. Figure 4 contains the histogram of measured LET values for the RW3‐I setup for both, experiments and simulations. In Section S8, the histograms for the other setups are reported. These histograms are split into two regions: a low‐LET range up to $25\;\mathrm{keV}\;\mu m^{-1}$ in Figure 4a and a high‐LET range with values greater than $25\;\mathrm{keV}\;\mu m^{-1}$ in Figure 4b, which is in logarithmic scale.

Considering the different ranges of the density values on the y‐axis, it is clear that the number of tracks in the low‐LET range was the main contribution in terms of fluence, with the high‐LET range containing less than 1% of the tracks for the simulations and the experiments. The solid line corresponds to the relative cumulative H values as a function of LET for both experiments and simulations. Tracks with low‐LET values contributed approximately 50% to 80% of the total H in the experimental and simulated data. As a result, a few high‐LET tracks that have a low probability of occurrence contributed on a relatively large scale to the total H. This can be seen by the relatively large fluctuations of the cumulative H in the high‐LET range, which reached 10% or more of the total H by a single track in the experiments. That effect was observed for the four setups, and higher LET values were present in the simulated data. The findings suggested that for comparing simulations with experiments, considering only the tracks in the low‐LET range might be more robust, while for the actual dose equivalent assessment, the low‐ and high‐LET ranges must be considered.

**FIGURE 5:**
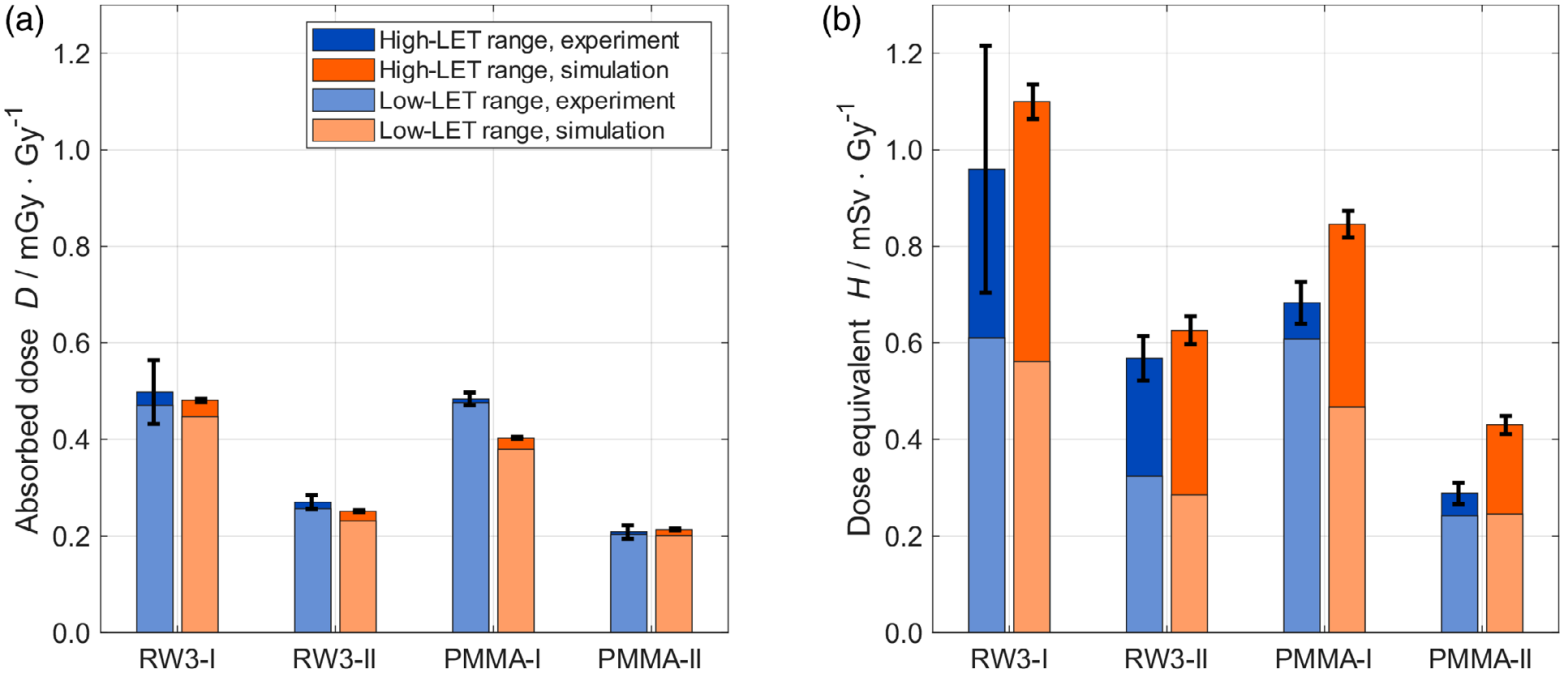
(a) Absorbed dose (D) and (b) dose equivalent (H) for the low‐LET range (light color) and high‐LET range (dark color) for the experimental measurements with FNTDs (blue) and the Monte Carlo (MC) simulations (orange).

In Figure 5, D and H values normalized to the target dose are reported, showing values for simulations and experiments. For D, a ratio between 0.8 to 1.0 for simulations over experiments was found. The contribution of the high‐LET particles was always found to be below 10%. A maximum D value of $\left(0.50\pm 0.07\right)\;\mathrm{mGy}\;{\mathrm{Gy}}^{-1}$ was found for position I and $\left(0.208\pm 0.014\right)\;\mathrm{mGy}\;{\mathrm{Gy}}^{-1}$ for position II. For H in the low‐LET range, agreement between simulations and experiments was observed, with ratios within 0.8 to 1.0. Instead, larger differences were found for the high‐LET range, with ratios up to a factor of 5. This is due to the high‐LET particles, as shown in Figure 4. However, comparing H values over the whole range, agreement within a factor of 1.1 to 1.5 was found. With the experiments, maximum H values of $\left(1.0\pm 0.3\right)\;\mathrm{mSv}\;{\mathrm{Gy}}^{-1}$ were measured for position I, and $\left(0.57\pm 0.05\right)\;\mathrm{mSv}\;{\mathrm{Gy}}^{-1}$ for position II, overall allowing for H estimation with FNTDs. For completeness, Figure S9 displays the out‐of‐field dose values without the range thresholding applied here.

### 3.4 Contribution of charged particles to the out‐of‐field dose equivalent

**FIGURE 6:**
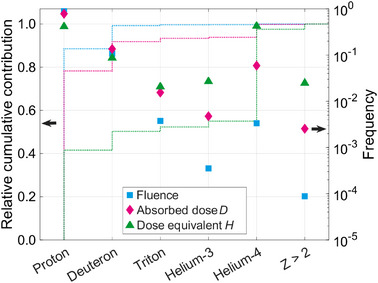
Contribution of each ion type to the fluence (cyan), D (magenta), and H (green) for the RW3‐I setup. The cumulative contribution is displayed with the dotted line on the left y‐axis. The relative contribution is represented by the markers on the right y‐axis using a logarithmic scale.

MC simulations were used to gather more detailed information on the measured signal. Figure 6 displays the relative frequency of the different ion types scored in setup RW3‐I, together with the relative cumulative contribution, showing the relative cumulative values of fluence, D, and H. Recoil protons were the main contributor to the total fluence, accounting for almost 90%. The second‐largest contribution was due to deuterons, accounting for about 10%. The other scored ions represented less than 1% of the fluence.

The relative contribution of each ion type to D and H is also displayed in Figure 6. Protons contributed about 80% to the total D, following the trend of the fluence. In contrast, both protons and helium‐4 contributed about 40% to the total H. However, helium‐4 only contributed about 5% to D, and less than 1% to the fluence. Deuterons represented the third largest contribution to H. The ion species triton, helium‐3, helium‐4, and heavier ions with Z>2 contributed almost one‐half of the total H, highlighting the disproportionate dosimetric impact of the high‐LET particles observed in the MC simulations, which have a low probability of occurrence. In other words, H is not mainly driven by the secondary protons. Further information on the energies and LET values of the observed charged particles is given in Section S10.

## 4 DISCUSSION

### 4.1 Methodological considerations for accurate dose equivalent determination

Accurate quantification of D and H with FNTDs requires several post‐processing steps, as demonstrated in this study. A key element is the correction for inter‐detector sensitivity variation, which can vary between a factor of 0.6to2.5 according to previous reports.^14^, ^27^ This is why a correction procedure using a reference irradiation with 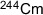 was adapted.^14^ The primary modification for this study was to perform the analysis in different areas and not in different depths, as the alpha particles used for sensitivity correction and the tracks from out‐of‐field radiation had a range overlap. This adjusted procedure might introduce some uncertainty in the sensitivity correction due to intra‐detector sensitivity variations, as the regions used for calibration and out‐of‐field signal evaluation can be spatially separated by up to 2mm. Nevertheless, a previous study found possible intra‐detector sensitivity variation of about 10% for such a spatial displacement,^23^ which is comparable small to the intrinsic inter‐detector sensitivity variation. As an advantage, this adjusted readout procedure allows for the compensation of any reader‐related performance changes, which can influence the measured signal when, for example, conducting the readout of the out‐of‐field signal and the 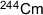 signal on different days.

Another important aspect identified in this study is the need for a polar angle–dependent correction of track spot intensity. To our knowledge, this is the first time that experimental data has been used to model the angular dependence of intensity decrease, using a generalized logistic function (Equation 1). The results shown in Figure 3 indicated a substantial decline in detected intensity for larger polar angles, with values dropping below 0.3 for angles exceeding $50^{\circ }$. This observation is consistent with findings from a previous study, where a similar trend between track polar angle and track spot intensity was reported, based on a theoretical model.^23^

Nevertheless, a direct comparison between the two models revealed notable discrepancies, particularly in the intermediate angular range of $20^{\circ }\;\mathrm{to}\;\;50^{\circ }$. For the model by Bartz et al.,^23^ an intensity reduction of approximately 52% was found at $30^{\circ }$, whereas the model presented here predicted a reduction to about 36%. Comparing these two values, the intensity calculated using Bartz's correction factors corresponds to only 70% of the intensity predicted by the model shown in Figure 3. These differences may lead to a relevant underestimation of intensity when applied to the data shown here. Such variation in correction factors might be due to differences in hardware components and optical configurations. This underscores the importance of both verifying and, if required, establishing a device‐specific correction model, such as the one performed here.

The methodology presented here determined D and H by analyzing particles that cross the scoring plane, implementing a 2D scoring approach, similar to that already done for PNTD.^8^, ^28^ The main reason for using this methodology with FNTDs rather than a 3D approach was that alumina has a relatively high mass density of $3.97\;g\;cm^{-3}$, resulting in a much shorter charged particle range compared to in water. Furthermore, alumina is non‐tissue equivalent; thus, the interaction between radiation and detector material is different from human tissue. This 2D approach allowed to consider only particles that enter the volume of interest, without being directly influenced by the detector material. Additionally, a minimum range of 10μm was imposed to allow track spot intensity averaging over several slices. These two assumptions inherently exclude tracks corresponding to high‐LET particles with shorter ranges. Such particles, however, do contribute substantially to H due to the amplification effect by the quality factor Q, as visualized in Figure 4. With regard to these short‐range particles, differences were observed between simulations and experiments. While in experiments, scoring short‐range particles resulted in H increasing up to 40%, simulations showed an increase of up to a factor of 5, highlighting a strong difference in these particles, as shown in Figure S9. Further investigation of these short‐range particles is required to enable a direct comparison with the default and built‐in volumetric DOSEQLET scorer in FLUKA.

### 4.2 Out‐of‐field measurements

In this study, experimental data have been obtained from out‐of‐field neutron measurements with FNTDs for a primary proton SOBP plan. Since the measurements were conducted behind the SOBP, it can be assumed that the measured radiation is predominantly due to neutrons generated in the phantom material. These results were used to benchmark MC data derived from FLUKA simulations. A comparison of the fluence revealed ratios of 1.2 to 1.3 for simulated values over experimental values. Differences between the implemented materials in the simulations and the actual materials used in the experiments could be responsible for this discrepancy, as well as challenges in detecting low‐LET particles and elongated track spots. Compared to previous studies where FNTD measurements were compared with the treatment planning system as reference, a ratio of almost 2 was found in the fluence.^24^ The main reason identified was the large proportion of particles with a LET value below $1\;\mathrm{keV}\;\mu m^{-1}$, which are rather challenging to detect.^29^ Since in this study, primary protons with a maximum energy of $172\;\mathrm{MeV}\;u^{-1}$ were used (see Table S2), the detection efficiency is assumed to be relatively high because only a few particles with an LET value below $1\;\mathrm{keV}\;\mu m^{-1}$ were present. A similar agreement was found for the polar angle of the tracks scored with both modalities. The deviations in the median angle in the order of a few degrees can be related to uncertainties originating from the positioning, either by a rotation during the irradiation or misalignment during the readout.

Experimental and simulated D values agreed, with simulation results within a factor of 0.8 to 1.0. For H values, larger variations were found, with results between a factor of 1.1 to 1.5 when considering the low‐ and high‐LET range (Figure 5). This deviation is primarily attributed to the contribution of the high‐LET particle tracks with LET values exceeding $25\;\mathrm{keV}\;\mu m^{-1}$, which are, on average, higher for the simulated data. For these particles, the amplification effect introduced by the quality factor, shown in Equation 3, further magnifies the difference between the simulation and the experiment, leading to larger H values for the simulations. Although these tracks only represented about 1% of the fluence, they can contribute more than 50% to the total H, as illustrated in Figure 4. Previous studies that utilized PNTD or Timepix detectors for the out‐of‐field measurements faced similar challenges when measuring H values, reporting discrepancies between simulations and experiments between 0.3to2.^30^, ^31^

Only a few experiments have previously been conducted under comparable geometric and treatment‐plan conditions. PNTDs were used to estimate the out‐of‐field neutron dose in a water phantom,^5^ yielding a H value of about $0.7\;m\mathrm{Sv}\;{\mathrm{Gy}}^{-1}$, which can be compared to the values obtained here. The distal position with regard to the isocenter was between the positions RW3‐I and RW3‐II of this study, for which H values of $\left(1.3\pm 0.5\right)\;\mathrm{mSv}\;{\mathrm{Gy}}^{-1}$ and $\left(0.59\pm 0.04\right)\;\mathrm{mSv}\;{\mathrm{Gy}}^{-1}$ were measured without range thresholding, respectively.

For practical applications, the question arises whether in‐phantom out‐of‐field dose estimations should rely solely on detector measurements or instead use these measurements to benchmark MC models, to be later employed for dose estimation. Both approaches have limitations; with passive detectors, only discrete positions can be analyzed, and an overview of the dose distribution cannot be obtained. With simulations, cross‐sections for neutron energies greater than 20MeV are scarce, resulting in neutron dose equivalent differences up to a factor of 3 when employing different models.^32^ Given that no single technique is mature enough for this purpose, a combined approach integrating both methods appear to be a reliable strategy for estimating out‐of‐field doses, as already done in the past.^30^, ^31^

### 4.3 Dosimetric impact of low‐probability events and large track angles

A main observation of this study was that while the out‐of‐field fluence is dominated by low‐LET particles with a value below $25\;\mathrm{keV}\;\mu m^{-1}$, this is not reflected in the contribution to the out‐of‐field H value. A major contribution to H comes from target fragments that are heavier than recoil protons, mainly helium‐4 (Figure 6). These results align with similar observations from space dosimetry, where studies revealed a large imbalance between relative contribution in terms of fluence and D or H when differentiating by the particle type, displaying a large dosimetric importance of scarce high‐LET ions.^33^ Conversely, for ions with a low atomic number, typically particles with a lower LET, detection might be less important because of the smaller biological importance relative to heavier ions. In any case, low probability events induce a challenge, because a large number of primaries must be simulated or a large detector area must be read out to observe these particles, thus achieving more reliable H values.

Another relevant aspect was the detection of elongated track spots associated with large polar angles. Tracks with polar angles exceeding $65^{\circ }$ present challenges in image processing,^34^ as demonstrated in Figure 2 and in the intensity correction illustrated in Figure 3. In the current experimental setup, where measurements were performed at distal positions behind the target volume, tracks with polar angles greater than $65^{\circ }$ were rare and made up less than 2% of the tracks. However, this proportion may increase in more clinically relevant scenarios, particularly for measurements at lateral positions. Moreover, according to Equation 2, such tracks have an increased dosimetric relevance due to their longer path lengths within the sensitive volume. Therefore, future studies should place greater emphasis on the detection and accurate analysis of these tracks.

### 4.4 Limitations of the MC model

Overall, the FLUKA model used in this study provided consistent results for physical quantities such as particle fluence, polar angle, and D. However, the accuracy of MC predictions, especially for high‐energy neutron fields, remains limited by the scarcity of experimental high‐energy neutron reference data. This was also expressed in previous studies, in which different MC codes and their results for high‐energy neutrons were compared, revealing relevant differences between them.^35^, ^36^ Furthermore, the limited number of high‐LET events scored in the MC simulations restricts their interpretability. As such, it is important to keep in mind the limitations of the MC simulation techniques for representing low probability events, as the ones strongly influencing H in out‐of‐field proton therapy. In principle, variance reduction techniques could be used to account for low probability events, but it is important to consider that variance reduction techniques need to be properly implemented for event‐by‐event scoring in MC simulations.

## 5 CONCLUSION

This study presented the first application of FNTDs to out‐of‐field in‐phantom secondary neutron dosimetry in proton beam therapy, establishing a methodology for measuring absorbed dose (D) and dose equivalent (H) based on particle surface‐crossing analysis. H determination was achieved by implementing a reliable assessment of the LET, inter‐detector sensitivity correction, and a polar angle–dependent correction to compensate for angle–dependent intensity variations.

Experimental measurements in phantoms of two different materials were in agreement with simulations, yielding simulated fluence values that differed by less than a factor of 1.3 from the experimental values. For H estimations considering only particles that can reach 10μm, a small fraction of high‐LET tracks contributed by almost 50%. These are mainly helium‐4 and ions with Z>2, which together account for about 1% of the total fluence. Simulation and experimental results for H agreed within a factor of 1.1 to 1.5, with a maximum measured H of $\left(1.0\pm 0.3\right)\;\mathrm{mSv}\;{\mathrm{Gy}}^{-1}$.

These results demonstrated that FNTDs, combined with the developed methodology, provide a viable approach for characterizing out‐of‐field neutron radiation fields in proton therapy, agreeing with MC simulations under the defined conditions. Further analysis of short‐range and high‐LET ions is needed to improve the quantification of their contribution to H.

## CONFLICT OF INTEREST STATEMENT

The authors declare no conflict of interest.

## Supporting information



Supporting information

## Data Availability

The data used in this study can be found in “Dataset for Fluorescent Nuclear Track Detectors for out‐of‐field neutron dosimetry in proton therapy”^37^ for the out‐of‐field data, and in “Calibration dataset for Fluorescent Nuclear Track Detectors for out‐of‐field neutron dosimetry in proton therapy”^38^ for the LET calibration and track polar angle correction.
