# Supplementary material for: Fluorescent nuclear track detectors for out‐of‐field neutron dosimetry in proton therapy
Source: Med Phys. 2026 Feb 10;53(2):e70303. doi: 10.1002/mp.70303 (PMC12888957; doi:10.1002/mp.70303)
Supplement: Supplementary file 1 — Supporting information [file MP-53-0-s001.pdf]

Supplementary Material

Fluorescent Nuclear Track Detectors for out-of-field neutron dosimetry in proton therapy

Stefan Schmidt[1,2,3,4], Iván D. Muñoz[1,2,3], Eduardo G. Yukihara[5], José Vedelago[1,2,3]

[1]Department of Radiation Oncology, Heidelberg University Hospital (UKHD), Im Neuenheimer Feld 400, 69120 Heidelberg, Germany
[2]Division of Medical Physics in Radiation Oncology, German Cancer Research Center (DKFZ), Im Neuenheimer Feld 280, 69120 Heidelberg, Germany
[3]Heidelberg Institute for Radiation Oncology (HIRO) and National Center for Radiation Research in Oncology (NCRO), Im Neuenheimer Feld 280, 69120 Heidelberg, Germany
[4]Medical Faculty Heidelberg, Heidelberg University, Im Neuenheimer Feld 672, 69120 Heidelberg, Germany
[5]Department of Radiation Safety and Security, Paul Scherrer Institute (PSI), Forschungsstrasse 111, 5232 Villigen, Switzerland

Contents

S1 **Material composition** **2**

S2 **Treatment plan validation** **3**

S3 **Sample Fluorescent Nuclear Track Detector images for out-of-field dose measurements** **6**

S4 **Linear energy transfer in water calibration curve** **7**

S5 **Parameters used for the Monte Carlo simulations** **9**

S6 **Poler angle threshold values for comparison between measurements and simulations** **10**

S7 **Validation of linear energy transfer in water values in mono-energetic neutron fields** **11**

S8 **Cumulative dose equivalent distribution** **13**

S9 **Evaluation of the minimum range requirement on the measured and simulated dose values** **15**

S10 **Energy and linear energy transfer in water distribution of simulated out-of-field particles** **16**

References **18**

S1 Material composition

Table S1: Material overview used in the Flukturierende Kaskaden (FLUKA) simulations. All materials were from the predefined FLUKA library. Polymethyl methacrylate (PMMA) and RW3 were defined according to Schoenfeld et al. (2015)[1,2].

Material name	Location	Mass fraction	Density / ($g \cdot cm^{-3}$)
Air	Irradiation room	C: 0.0001248; N: 0.755267; O: 0.231781; Ar: 0.012827	0.0012
Concrete	Room walls	H: 0.01; C: 0.001; O: 0.529107; Na: 0.016; Mg: 0.002; Al: 0.033872; Si: 0.337021; K: 0.013; Ca: 0.044; Fe: 0.014	2.3
Mylar	Exit window	H: 0.042; C: 0.6251; O: 0.3329	1.4
PMMA	Detector holder, Phantom	H: 0.0804; C: 0.6; O: 0.3196	1.19
RW3	Phantom	H: 0.0759; C: 0.9041; O: 0.008; Ti: 0.012	1.045
Tungsten	BAMS	W: 1.00	19.3
Water	BAMS	H: 0.1119; O: 0.8881	1.0

S2 Treatment plan validation

In Table S2, the treatment plan used in this study is tabulated, with the iso-energy slices (IES), the energies, as well as the focus and the number of primaries per slice. Each slice contains 1089 raster points. The number of primaries in each IES represents the total number of primaries to achieve a 1 Gy spread-out Bragg peak (SOBP) in a water phantom.

In Figure S1, the Monte Carlo (MC) simulated treatment plan is shown, verifying the target cube in water, as well as showing the dose distribution when using a RW3 or a polymethyl methacrylate (PMMA) phantom. Both a 2D profile for the x-z plane as well as a depth dose profile for the z-axis are shown. The treatment plan was initially designed for a water phantom and was not adjusted for the other phantom materials to maintain consistency in the number of primaries and energies used. The average absorbed dose in the MC simulations for the water phantom in the SOBP is approximately 0.97 Gy, revealing a nominal discrepancy of about 3 % to the reference dose. For the RW3 phantom material, shown in Figure S1b) and e), the dose distribution is similar to the one in water, even though the depth dose profile is shifted by less than 1 cm to the proximal side of the phantom. Additionally, the peak dose is below that for water, resulting in 0.94 Gy. For PMMA, for which the density of the material is about 19 % higher compared to that of water, the SOBP is shifted and squeezed towards the proximal side of the phantom, being within -6.4 to 1.6 cm. The absorbed dose at the SOBP is about 0.94 Gy. The lateral extension of the field is comparable between the different phantom materials. Figure S1 also highlights the end of the range of the protons, resulting in a line-shape structure with a dose drop right behind the SOBP. The residual dose beyond the range is mainly neutron-induced.

Table S2: Treatment plan for a 10 cm SOBP in 10 cm depth of a water phantom. The number of primaries results in 1 Gy target dose in water.

IES number	Energy / $MeV \cdot u^{-1}$	Focus / mm	Primaries per IES $\cdot 10^9$
1	118.78	13.5	0.79
2	120.69	13.3	0.64
3	121.95	13.2	0.51
4	123.82	13.0	0.80
5	125.67	12.9	0.77
6	127.51	12.7	0.80
7	129.32	12.6	0.84
8	131.11	12.5	0.86
9	132.89	12.3	0.88
10	134.65	12.2	0.90
11	136.39	12.1	0.94
12	138.12	12.0	1.12
13	140.41	11.8	1.40
14	141.54	11.7	0.60
15	143.78	11.6	1.69
16	144.90	11.5	0.51
17	147.11	11.4	2.13
18	149.86	11.2	2.00
19	151.50	11.1	1.04
20	153.12	11.0	1.81
21	154.74	10.9	1.47
22	156.35	10.8	1.90
23	157.96	10.7	1.82
24	159.56	10.6	2.14
25	161.15	10.5	2.26
26	162.74	10.4	2.57
27	164.32	10.3	2.87
28	165.89	10.2	3.25
29	167.46	10.1	3.25
30	168.50	10.1	3.25
31	170.05	10.0	3.96
32	171.60	9.9	21.84

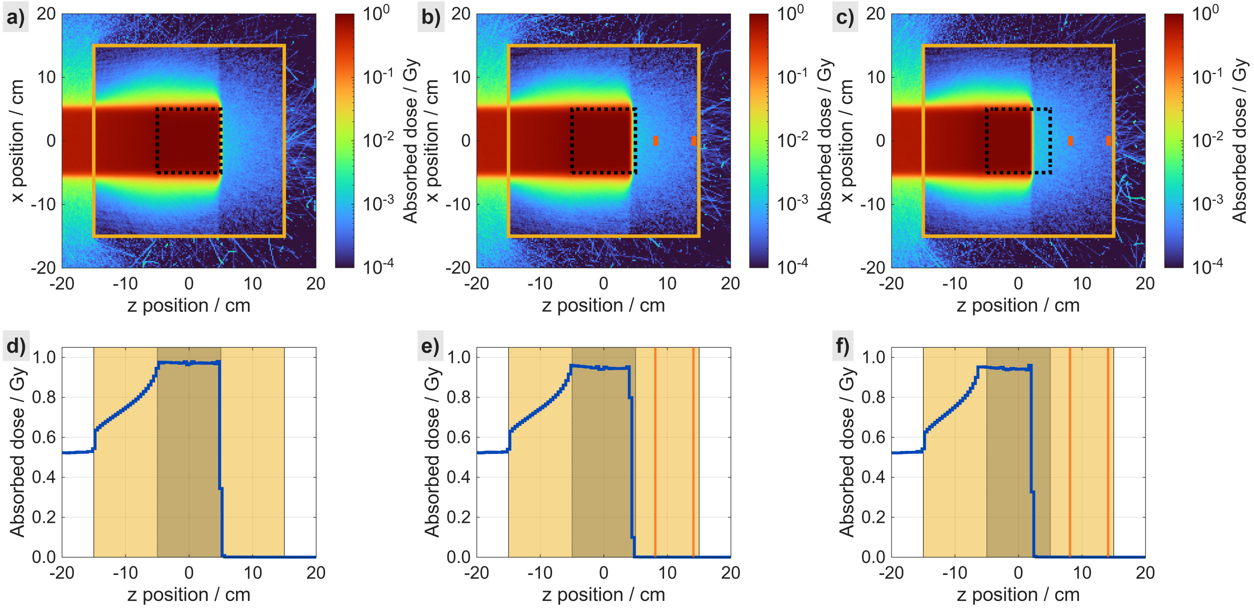

Figure S1: Panels a–c show 2D dose distributions averaged over the y-coordinate in the range −3 cm to 3 cm. In these images, the yellow area indicates the phantom, the black dotted box marks the planned target volume, and the orange boxes indicate the measurement positions. Panels d–f show the corresponding 1D depth dose profiles along the z-axis, with both x and y averaged over the range −3 cm to 3 cm. Panels a) and d) correspond to the water phantom, b) and e) to RW3, and c) and f) to PMMA.

S3 Sample Fluorescent Nuclear Track Detector images for out-of-field dose measurements

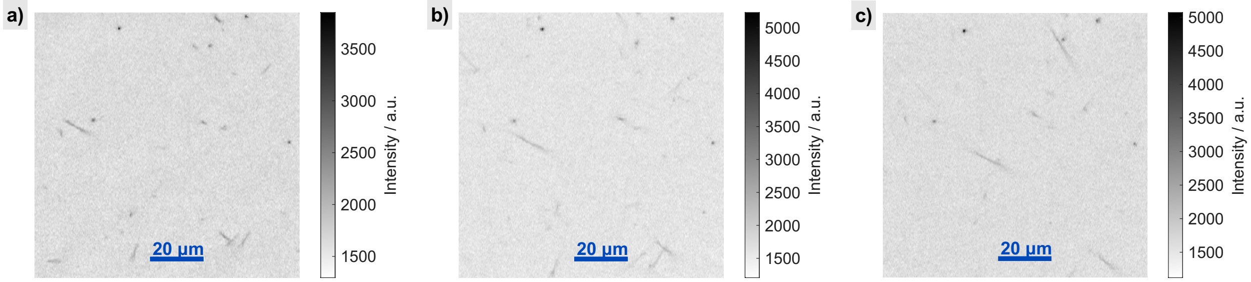

Figure S2: Illustration of a Fluorescent Nuclear Track Detector (FNTD) microscope image for the out-of-field dose measurement with the RW3-I setup. Images at different depths of the FNTD are displayed, namely a) 2 µm, b) 10 µm, and c) 20 µm.

S4 Linear energy transfer in water calibration curve

To perform linear energy transfer in water (LET)-based measurements of absorbed dose and dose equivalent, an LET calibration curve is required that relates the measured track-spot intensity to the corresponding LET. The experimental data were obtained from measurements of 5 MeV protons from the Ruđer Bošković Institute (RBI; Zagreb, Croatia), by 10 MeV and 13.8 MeV proton irradiations at the Physikalisch-Technische Bundesanstalt (PTB; Braunschweig, Germany), and by measurements at the Heidelberg Ion Beam Therapy Center (HIT; Heidelberg, Germany). Here, measurements with nominal proton energies of 48.12 MeV u^{-1} and 221.06 MeV u^{-1}, as well as 88.83 MeV u^{-1} ^{12}C ions, and 103.77 MeV u^{-1} ^{16}O ions, were conducted. For each measurement point, a total of four Fluorescent Nuclear Track Detectors (FNTDs) were irradiated. The readout was conducted with similar parameters as previously presented, but with 49 readout fields per detector (corresponding to an area of 0.0048 cm^2), and with 12 slices in depth, from 2 µm to 24 µm. Table S3 provides an overview of the used ions, nominal energies, corrected energies, and corresponding LET values. The corrected energies consider the energy loss from the source to the measurement position. For the measurements at PTB and RBI, for which the irradiations were conducted in a

Table S3: Overview of primary particles, their energies, corrected energies, and corresponding linear energy transfer in water (LET) values used to establish the intensity-to-LET calibration curve. The corrected energy accounts for the energy loss of the primary particles up to the scoring plane inside the detector.

Facility	Ion type	Nominal energy / MeV u^{-1}	Corrected energy / MeV u^{-1}	LET / keV µm^{-1}
HIT	^{1}H	221.06	219.95	0.43
HIT	^{1}H	48.12	44.67	1.37
PTB	^{1}H	13.80	13.69	3.61
PTB	^{1}H	10.00	9.86	4.75
RBI	^{1}H	5.00	4.75	8.51
HIT	^{12}C	88.83	82.33	30.60
HIT	^{16}O	103.77	96.04	48.50

vacuum chamber, only the attenuation of 10 µm in alumina was considered, accounting for the readout range between 2 µm to 24 µm. For the measurements at HIT, 950 µm water and 6 µm tungsten for the Beam Application and Monitoring System (BAMS), 1.5 m of air and 10 µm of aluminium oxide were considered for the energy loss calculation. The calculations were performed using the atomic interaction with matter (ATIMA) software, employing the continuous slowing-down approximation (CSDA) [3].

Based on these experimental data, a previously proposed logarithmic model [4] was fitted to the data points, according to Equation 1:

$$y = a \cdot \log_{10}\left(\frac{\mathrm{LET}}{b} + 1\right) \tag{1}$$

where y is the measured track intensity, and a and b are two fitting parameters. By applying a nonlinear regression, values of $a = (4840 \pm 110)$ a.u. and $b = (1.08 \pm 0.07)$ keV µm^{-1} were obtained, resulting in a coefficient of determination of 0.999 (see Figure S3).

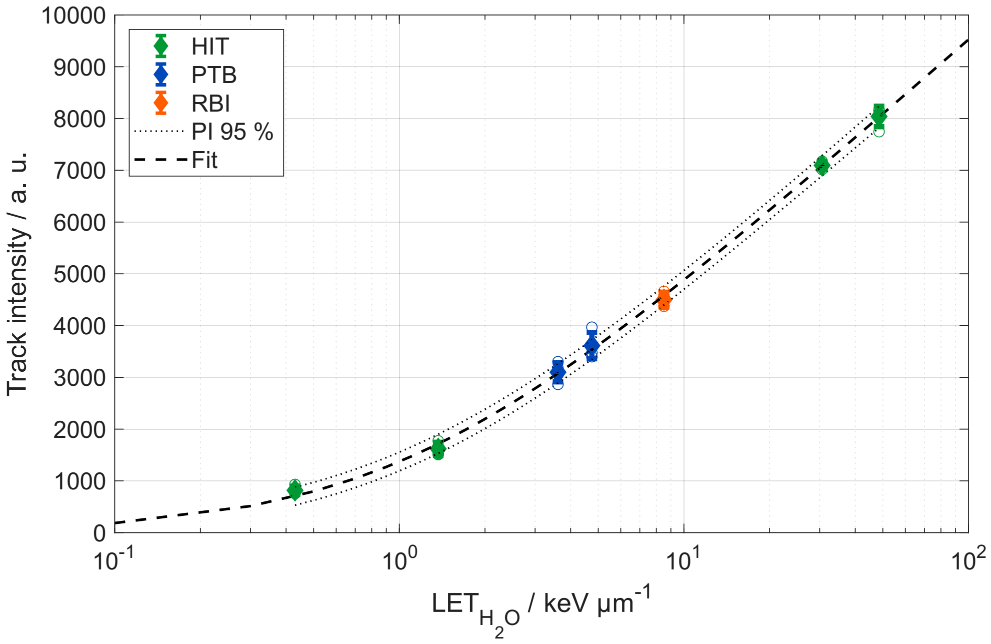

Figure S3: Visualization of the LET calibration curve used in this study, which converts measured track intensity into LET. The calibration was established using measurements from three institutes: HIT for protons, carbon ions, and oxygen ions, RBI for protons, and PTB for protons.

S5 Parameters used for the Monte Carlo simulations

Table S4: Summary of the Monte Carlo (MC) simulation parameters, formatted according to the American Association of Physicists in Medicine (AAPM) Task Group 268 recommendations[5].

Item	Description
MC code	FLUKA 4-5.0 and FLAIR 3.4-0 versions[6,7,8].
Validation	Comparison with measured fluence, polar angle distribution, physical dose, and dose equivalent using FNTDs. Depth–dose profiles for a water phantom were compared with treatment planning system expectations, as shown in Figure S1.
Timing	500 CPU cores with calculation times up to 72 hours per core, executed on the BWUniCluster 3.0.
Geometry	Simplified Beam Accelerator Monitoring System and cubic phantom geometry. The surrounding room was modeled as a rectangular enclosure[9].
Materials	Materials were selected based on the literature or FLUKA's built-in materials, as summarized in Table S1[1].
Source description	Parallel primary proton beams of 10 cm × 10 cm. Energies sampled from 118.78 MeV u^{-1} to 171.60 MeV u^{-1} based on the treatment plan using a custom source routine. The treatment plan with iso-energy slices is shown in Table S2.
Cross-sections	JEFF-3.3 libraries and PEANUT model[10,11,12].
Transport parameters	PRECISIOn setting with physics cards for evaporation and coalescence. IONTRANS was fully activated. Electromagnetic field (EMF) effects are deactivated.
Scored quantities	Customized mgdraw user routine to score particle type, kinetic energy (MeV), linear energy transfer in water (LET, keV μm^{-1}), polar angle (°), and direction; USRBIN used to visualize 1D and 2D absorbed dose distributions.
#histories	For the main simulations with different phantom materials, $2 \cdot 10^8$ primaries per spawn were simulated, with 500 spawns in total.
Postprocessing	Absorbed dose and dose equivalent calculated offline from particle fluence and LET.

S6 Poler angle threshold values for comparison between measurements and simulations

To enable consistent comparison between simulations and experimental results, range thresholds were applied to the experimental data to ensure accurate signal detection and effective separation of delta electrons from recoil protons and fragments. In the simulations, results were scored on a plane, capturing the particles' angles and kinetic energies but not their ranges. Based on the particle's polar angle and the threshold depth of 10 µm in alumina, the required range of the particle can be calculated. Using the calibration curves in Figure S4, the required kinetic energy can be calculated and compared to the actual kinetic energy of the particles. If the energy of the particle is not sufficient, it is not considered. The data for these calibration curves were taken from ATIMA[3]. The curves in Figure S4 were power-law modeled and established for six different charged particles, namely proton, deuteron, triton, helium-3, helium-4, and charged particles with $Z > 2$. For the latter, the calibration curve was determined for carbon ions.

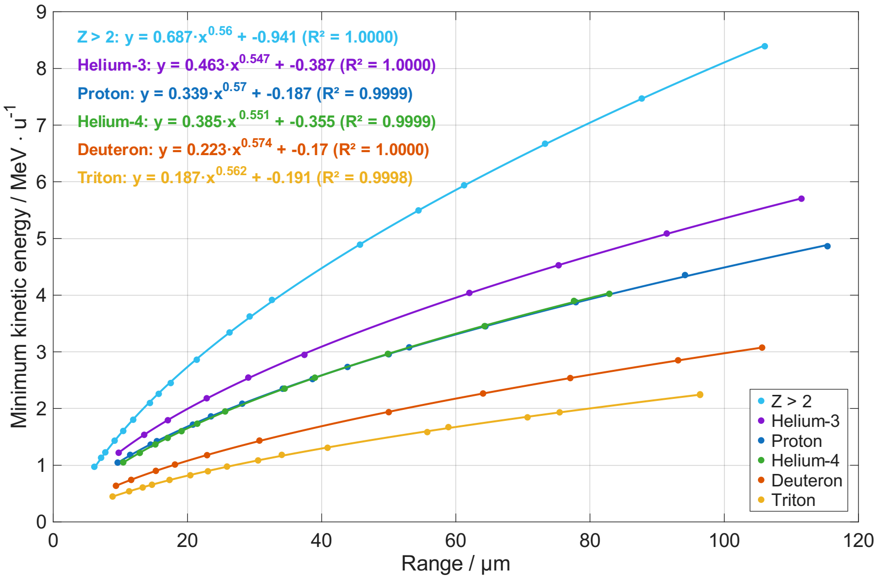

Figure S4: Particle-specific functions used to convert the range value, determined by the track's polar angle required to reach 10 µm depth, into the corresponding minimum kinetic energy.

S7 Validation of linear energy transfer in water values in mono-energetic neutron fields

The capability of determining LET values from track intensities was evaluated using two mono-energetic neutron fields of 6.5 MeV and 19 MeV, measured at the PTB, as reported previously[2]. A bin width of 1 keV µm^{-1} was selected for the LET distributions, with intervals defined as [0, 0.5), [0.5, 1.5), [1.5, 2.5), and so on. The experimentally obtained spectra, derived from three different FNTDs, were compared to the simulated spectra by analyzing both the median LET values and the interquartile range (Q1–Q3).

For the 6.5 MeV neutron field, the median LET was approximately 11.5 keV µm^{-1} in simulations, while experimental values ranged between 8.1 keV µm^{-1} to 10.6 keV µm^{-1}. This discrepancy is likely due to the limited ability of the experimental setup to detect high-LET recoil protons, whose ranges are typically on the order of a few micrometres. In contrast, the simulations do not suffer from such detection limitations and can fully account for short-range, high-LET events. For the 19 MeV neutrons, the simulated median LET was 4.5 keV µm^{-1}, while the experimental values ranged from 4.9 keV µm^{-1} to 6.8 keV µm^{-1}. A distinct peak at 4 keV µm^{-1} was observed in the simulations but appeared attenuated in the experimental data. Additionally, although no tracks with LET values below 3 keV µm^{-1} were observed in the simulations, a small number were detected experimentally, accounting for less than 5 % of the total track population. These low-LET tracks may be attributed to false positives from delta electrons, as also evident in the 6.5 MeV data.

Overall, the data demonstrated reasonable agreement between simulations and experiments for the 19 MeV neutron field. The larger discrepancy observed for 6.5 MeV neutrons is likely due to the reduced detection efficiency for short-range protons, which are more prevalent at lower neutron energies. Additional sources of uncertainty included corrections applied to the track-polar angle-to-intensity conversion, calibration of the LET-intensity relationship, and fluctuations arising from energy-loss straggling. Intra-detector sensitivity variations also contributed to the overall measurement uncertainty.

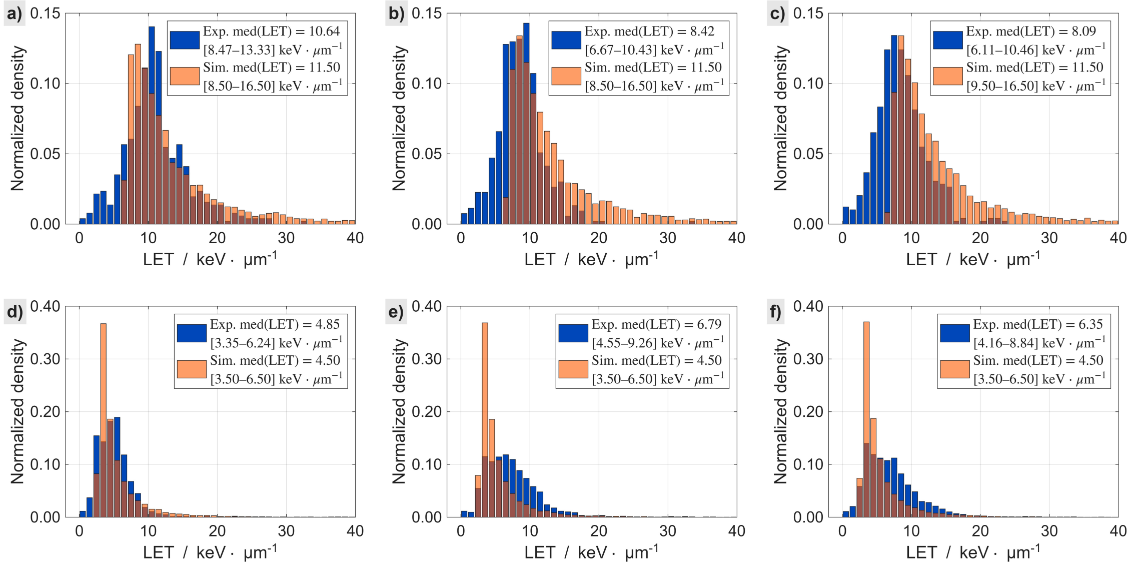

Figure S5: Overview of LET value determination, using the recoil proton spectra from mono-energetic neutron irradiations, with panel a), b), and c), showing the data for three FNTDs for 6.5 MeV neutrons, and panel d), e), and f), displaying the data for three FNTDs irradiated with 19 MeV neutrons. The values are compared to data from MC simulations.

S8 Cumulative dose equivalent distribution

The following figures present the contributions to the total dose equivalent as a function of LET, in the same format as Figure 4. Figure S6 presents the results for the measurement setup RW-II. Figures S7 and S8 present the corresponding results for the PMMA-I and PMMA-II setups, respectively.

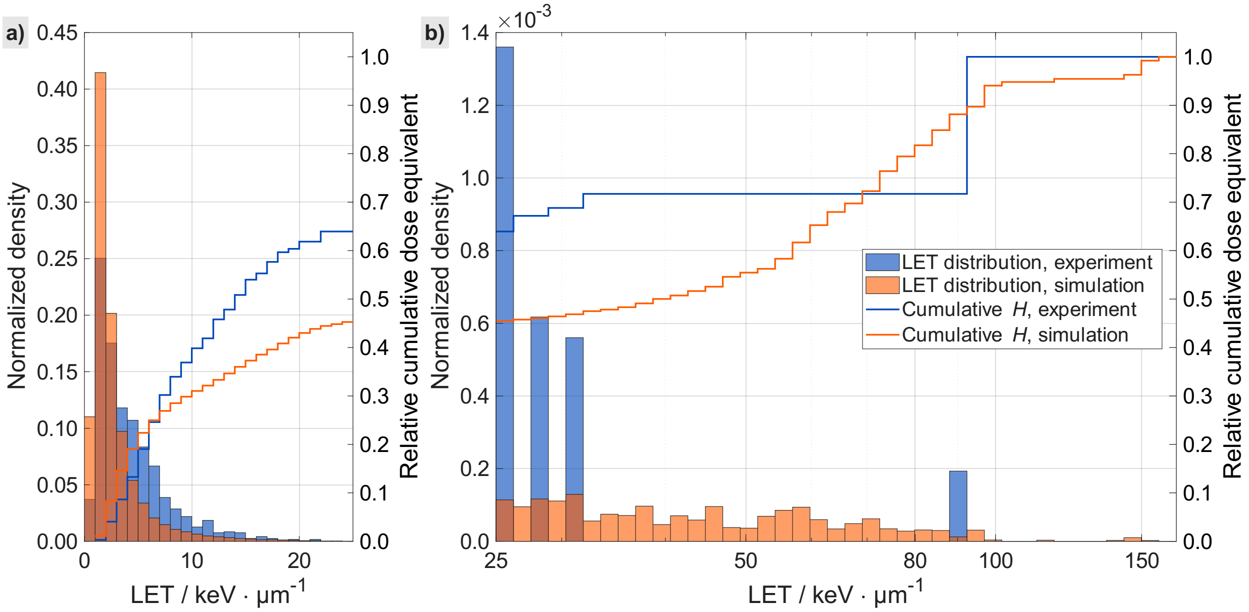

Figure S6: LET value distribution and cumulative dose equivalent curve for the setup RW3-II, for the low-LET range in a) and the high-LET range in b).

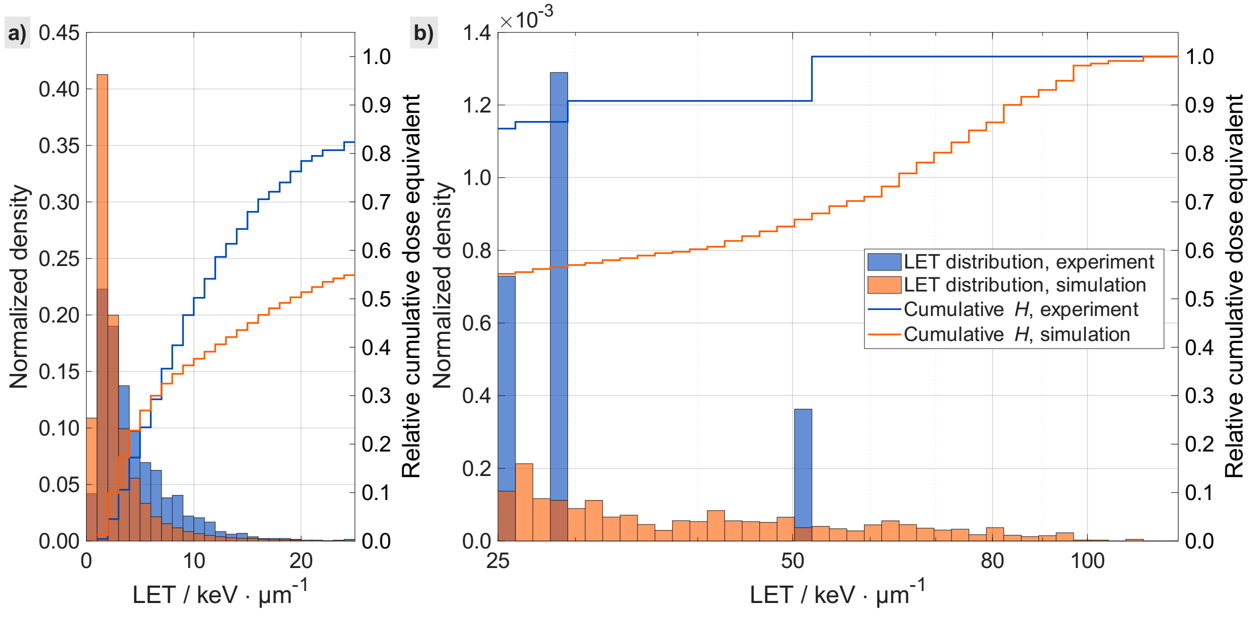

Figure S7: LET value distribution and cumulative dose equivalent curve for the setup PMMA-I, for the low-LET range in a) and the high-LET range in b).

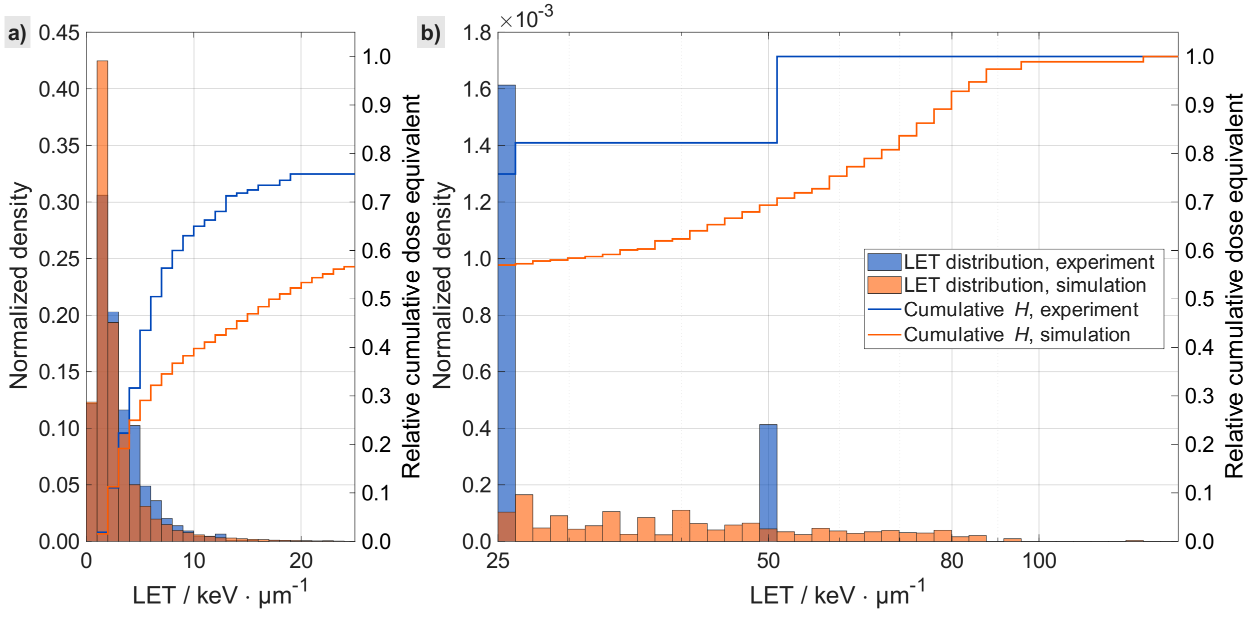

Figure S8: LET value distribution and cumulative dose equivalent curve for the setup PMMA-II, for the low-LET range in a) and the high-LET range in b).

S9 Evaluation of the minimum range requirement on the measured and simulated dose values

Evaluation of absorbed dose and dose equivalent values for particles regardless of their range. While absorbed dose values agreed within a factor of 1.2 to 1.4 between simulations and experiments, differences of a factor of 4 to 6 were observed for dose equivalent values. The particles with a range of shorter than 10 µm contributed by about 1 % and 5 % to the total fluence in simulations and experiments, respectively.

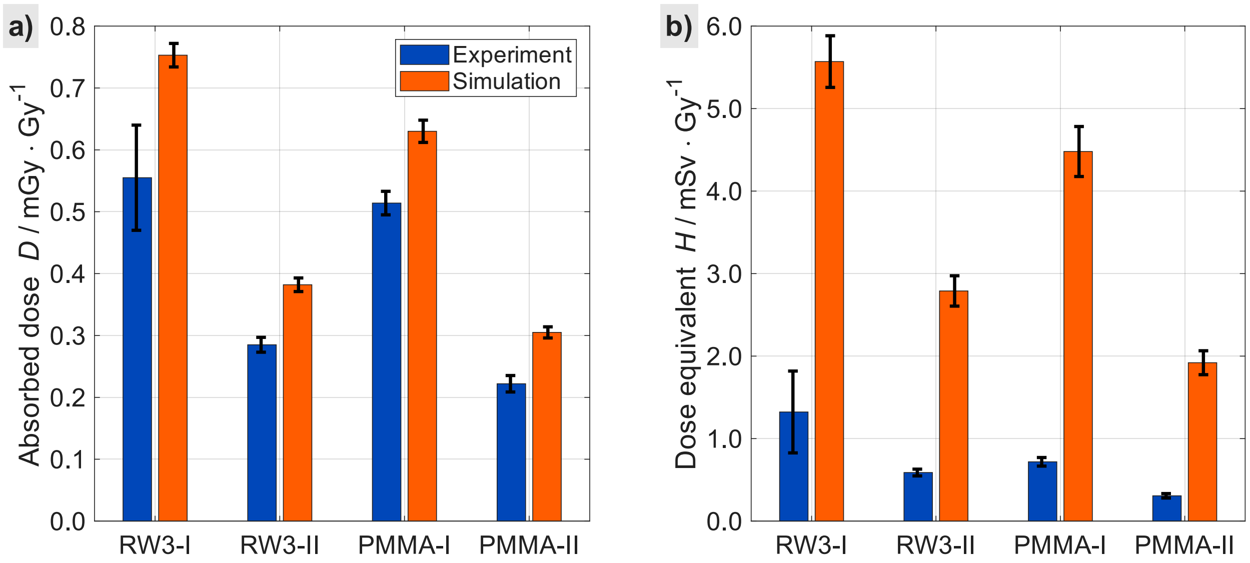

Figure S9: a) Absorbed dose D and b) dose equivalent H values for the experimental measurements and the MC simulations considering particles with all LET values and particles with all ranges.

S10 Energy and linear energy transfer in water distribution of simulated out-of-field particles

Analysis of additional field information for the RW3-I setup, applying the minimal range restriction. In Figure S10, the energy distribution, separated by charged-particle type, is shown in units of lethargy. Figure S11 depicts the LET value distribution for the different charged particles scored, for the low LET range up to 25 keV μm^{-1} in Figure S11a and above in Figure S11b. The polar angle distribution of the different charged particle types scored is displayed in Figure S12.

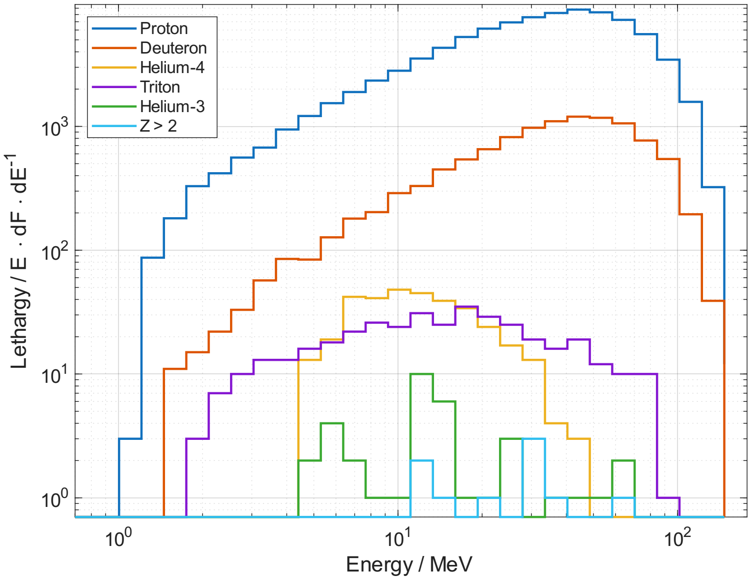

Figure S10: Energy spectrum for the different charged particles scored in the RW3-I setup. The y-axis is shown in units of lethargy.

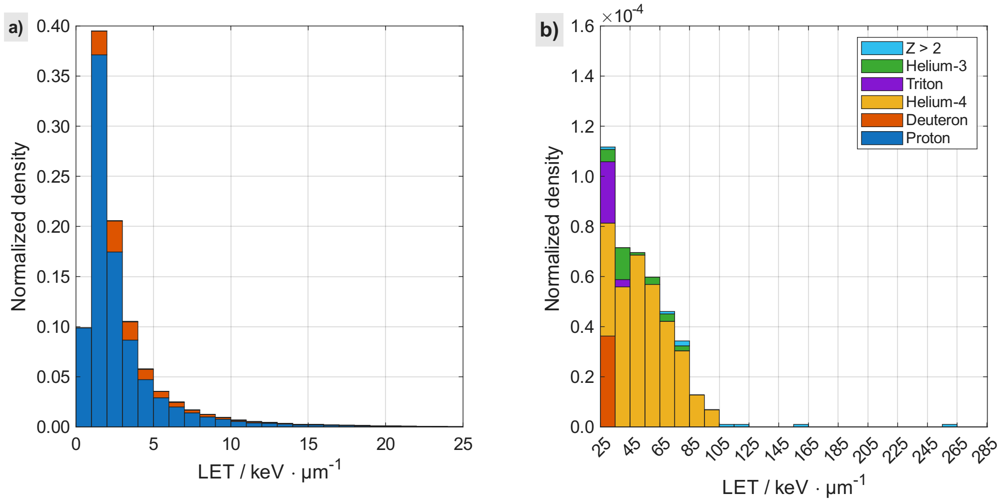

Figure S11: LET values of the measured charged particles in the simulation for the RW3-I setup, separated into a) the low and b) the high-LET range.

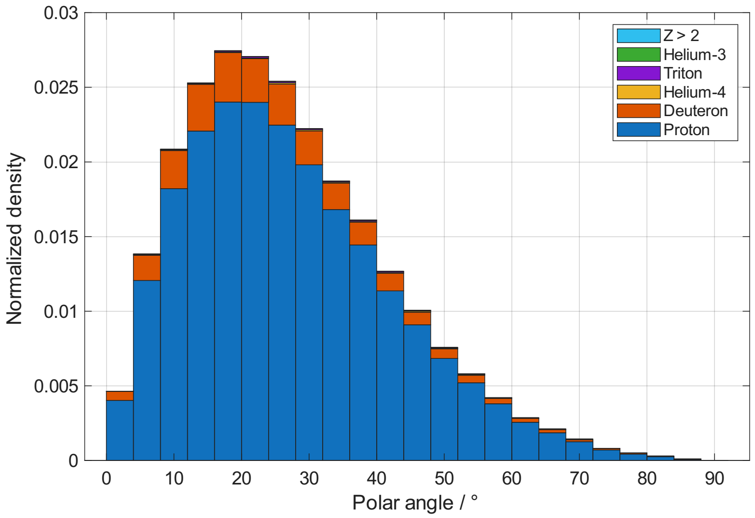

Figure S12: Angular distribution of scored charged particles in the RW3-I setup. Contribution is separated for the different charged particles.

References

[1] A. A. Schoenfeld, D. Harder, B. Poppe, and N. Chofor, Water equivalent phantom materials for 192Ir brachytherapy, PHYS MED BIOL **60**, 9403–9420 (2015). doi: 10.1088/0031-9155/60/24/9403.

[2] S. Schmidt, J. B. Christensen, B. Lutz, A. Stabilini, E. G. Yukihara, and J. Vedelago, Sensitivity analysis of fluorescent nuclear track detectors for fast and high-energy mono-energetic neutron dosimetry, MED PHYS **52**, e17799 (2025). doi: 10.1002/mp.17799.

[3] H. Geisel, C. Scheidenberger, P. Malzacher, J. Kunzendorf, and H. Weick, WebAtima - Energy Loss Calculator, Accessed: 2025-08-04. URL: https://www.isotopea.com/webatima.

[4] I. D. Muñoz, L. N. Burigo, T. Gehrke, S. Brons, S. Greilich, and O. Jäkel, Sensitivity correction of fluorescent nuclear track detectors using alpha particles: Determining LET spectra of light ions with enhanced accuracy, MED PHYS **50**, 2385–2401 (2022). doi: 10.1002/mp.16083.

[5] I. Sechopoulos, D. W. O. Rogers, M. Bazalova-Carter, et al. RECORDS: improved Reporting of montE CarlO RaDiation transport Studies: Report of the AAPM Research Committee Task Group 268, MED PHYS **45**, e1–e5 (2017). doi: 10.1002/mp.12702.

[6] G. Battistoni, T. Boehlen, F. Cerutti, et al. Overview of the FLUKA code, ANN NUCL ENERGY **82**, 10–18 (2015). doi: 10.1016/j.anucene.2014.11.007.

[7] C. Ahdida, D. Bozzato, D. Calzolari, et al., New Capabilities of the FLUKA Multi-Purpose Code, AIP CONF PROC **9**, 788253 (2022). doi: 10.3389/fphy.2021.788253.

[8] A. Donadon, G. Hugo, C. Theis, and V. Vlachoudis, FLAIR3–recasting simulation experiences with the Advanced Interface for FLUKA and other Monte Carlo codes, in *EPJ Web of Conferences* **302**, 11005 (2024). doi: 10.1051/epjconf/202430211005.

[9] K. Parodi, A. Mairani, S. Brons, et al. Monte Carlo simulations to support start-up and treatment planning of scanned proton and carbon ion therapy at a synchrotron-based facility, PHYS MED BIOL **57**, 3759–3784 (2012). doi: 10.1088/0031-9155/57/12/3759.

[10] E. Mendoza, D. Cano-Ott, C. Guerrero, and R. Capote, New evaluated neutron cross section libraries for the GEANT4 code, (2012). doi: 10.61092/iaea.6zqr-818y.

[11] E. Mendoza, D. Cano-Ott, T. Koi, and C. Guerrero, New Standard Evaluated Neutron Cross Section Libraries for the GEANT4 Code and First Verification, IEEE TRANS NUCL SCI **61**, 2357–2364 (2014). doi: 10.1109/tns.2014.2335538.

[12] E. Mendoza and D. Cano-Ott, Update of the Evaluated Neutron Cross Section Libraries for the Geant4 Code, (2018), INDC(NDS)–0758 INIS. doi: 10.61092/iaea.5knd-4xdd.
